# Effects of nitrogen application rate and weak light post anthesis on the grain yield and starch physicochemical properties of soft wheat

**DOI:** 10.3389/fpls.2025.1543407

**Published:** 2025-03-25

**Authors:** Tingting Yang, Abdul Rehman, Suhui Yan, Juan Chen, Jing Li, Xiao Zhang, Wenyang Li

**Affiliations:** ^1^ Anhui Province Key Laboratory of Functional Agriculture and Functional Food, Agronomy College of Anhui Science and Technology University, Fengyang, China; ^2^ Key Laboratory of Wheat Biology and Genetic Breeding in the Middle and Lower Reaches of the Yangtze River, Lixiahe Institute of Agricultural Sciences in Jiangsu, Yangzhou, China

**Keywords:** nitrogen application rate, weak light, soft wheat, yield, starch content, starch physicochemical properties

## Abstract

This study examined the effects of nitrogen (N) application rates and weak light treatment post anthesis on the grain yield and starch physicochemical characteristics of soft wheat. The soft wheat varieties Quanmai 725 (QM725) and Yangmai 15 (YM15) were used as study materials under field conditions, and the experiments were conducted during 2022–2023. During the grain filling stage (7–35 days post anthesis), three shading levels were set: 10% shading (S1), 20% shading (S2) and 30% shading (S3), with natural light conditions used as the control (CK). In 2023–2024, two N application rates (120 kg/hm^2^ [N1] and 180 kg/hm^2^ [N2]) and the abovementioned three shading treatments for each N application rate were set during the filling stage. The effects of weak light treatment post anthesis on the grain yield and yield components of soft wheat were analyzed. Moreover, the mitigation effects of different N application rates on the grain yield and starch physicochemical characteristics of wheat were examined. The results showed that N application increased wheat yield and yield components as well as the content of starch and its components, whereas weak light treatment decreased these parameters under the same N application rate. Under N1 and N2 conditions, weak light treatment post anthesis significantly reduced the volume, surface area percentage and number of B-type starch granules (particle size ≤10 μm) and increased those of A-type starch granules (particle size >10 μm). Enhanced N application rates significantly improved the gelatinization characteristics and thermodynamic characteristics of wheat starch. Under the same conditions of N1 and N2, weak light treatment significantly reduced the gelatinization characteristics of wheat starch, such as peak viscosity, trough viscosity and final viscosity. Although the enthalpy of wheat starch was increased, its onset temperature, peak temperature and end temperature were significantly reduced, which affected the quality of wheat grains and eventually led to a decrease in wheat yield. However, enhanced N application rates increased the grain yield and starch physicochemical characteristics of wheat. Under the same N application rate, weak light treatment post anthesis reduced the content of starch and its components in wheat grains, which in turn affected the wheat grain weight. The effect was more pronounced in wheat B-type starch granules than in A-type starch granules.

## 1 Introduction

Wheat (*Triticum aestivum L.*) is one of the most important food crops in China. The planting area of wheat accounts for approximately 20% of the total area of food crops (Yan et al., 2024). According to statistical data, 35%–40% of the world’s population feeds on wheat (Mottaleb et al., 2023). Furthermore, the demand for wheat is expected to increase at a rate of 1.6% per year by 2050 (Singh et al., 2016; Liu et al., 2022). Owing to frequent human activities, atmospheric turbidity and increase in aerosol concentration, frequent rainy weather has a serious impact on agricultural production and food security (Mastilović et al., 2018). These adverse effects have led to a 35% reduction in the amount of photosynthetically active radiation reaching the surface, resulting in a significant reduction in crop yield (Bergin et al., 2001). Long-term weak light not only leads to a decrease in temperature but also potentially causes a series of interannual climate changes that affect the global ecosystem (Upadhyay, 2020). Climatic and ecological factors, particularly light conditions, have become limiting factors for increasing crop yield posing a major challenge for crop production worldwide (Thies et al., 2024).

Nitrogen (N) is an essential nutrient element in the growth and development of wheat, which critically influences the grain yield and quality of wheat. Moreover, the absorption of N is closely related to light intensity (Maqsood et al., 2014). The regulation of N fertilizer application is not only the key to improving grain yield but also an important measure to enhance grain quality (Mandic et al., 2015). Weak light treatment post anthesis primarily affects the transport of stored N in the vegetative organs of wheat, increases the contribution rate of stored N before anthesis to grain yield and influences grain quality (Aguirre et al., 2006). Insufficient light during the grain filling stage not only decreases the accumulation of photosynthetic substances, grain filling rate and grain starch content but also reduces wheat starch synthesis and accumulation. The effect of N on wheat starch quality is more pronounced than that on wheat yield (Asthir et al., 2017), and different wheat varieties exhibit varying adaptability to weak light in terms of grain yield and starch quality. In wheat grains, appropriate N application rates may increase the content of starch and its components, and the effect of N on amylopectin content was less pronounced than that on amylose content (Khan and Akma, 2021). In the current agricultural production and application, excessive application of N fertilizers is frequently performed to improve crop yield, which also causes serious harm to the ecological environment (Hitz et al., 2017). Therefore, under weak light treatment, reasonable application of N fertilizers has become the key to improving crop yield and N use efficiency (Randhawa et al., 2017).

The Jianghuai wheat region in Anhui is located between the Yangtze River and Huaihe River (Zhang et al., 2020). Due to the influence of environmental factors such as rainy and humid conditions during the grain filling stage, the accumulation efficiency of grain proteins is reduced, resulting in low protein content (Wang et al., 2023). These climatic and environmental conditions indirectly provide suitable conditions for the production of soft wheat; moreover, they are suitable for the development of high-quality biscuits and pastry-soft wheat-producing areas (Souza et al., 2012). The grain yield enhancement technology in Jianghuai region has made substantial progress; however, due to the impact of climate change, agricultural production remains unstable, and it is difficult to adapt to the demands of large-scale new agricultural production and the supply of high-quality soft wheat (Du et al., 2019). Current studies on the effects of N and weak light treatment on wheat have mostly focus on single-factor effects, such as dry matter, yield and quality. There are relatively few studies on the multi-factor effects of N application rate and weak light treatment after flowering on the grain yield and starch physicochemical characteristics of soft wheat. Therefore, we examined the high-yield and high-efficiency cultivation of wheat using the soft wheat varieties Quanmai 725 (QM725) and Yangmai 15 (YM15) as study materials. By setting different N application rates and light levels, the differences in the grain yield and quality of soft wheat under different N application rates and weak light stress were determined. The relationship between wheat yield and quality under N fertilizer application and weak light treatment was examined to provide a theoretical basis and technical approach for alleviating the adverse effects of weak light stress on wheat yield and quality post anthesis in the Jianghuai region.

## 2 Materials and methods

### 2.1 Experimental design

Field experiments were conducted in the plantation area (32°52′31′′N, 117°33′52′′E) of Anhui Science and Technology University in Fengyang county, Anhui province over two consecutive growing seasons—from 2022 to 2023 and from 2023 to 2024. The test materials included the soft wheat varieties QM725 and YM15. The previous crop was corn, and the planting density was 1.8 million plants/hm^2^. The soil organic matter content in the plough layer (0–20 cm) of the test site was 16.65 g·kg^−1^, alkali-hydrolysable N content was 72.75 mg·kg^−1^, available potassium content was 96.05 mg·kg^−1^ and available phosphorus content was 17.45 mg·kg^−1^. The amounts of N, phosphorus and potassium fertilizers applied were 180 kg N hm^2,^ 90 kg P_2_O_5_ hm^2^ and 90 kg K_2_O hm^2^, respectively. Phosphorus and potassium fertilizers were applied as base fertilizers. For N fertilizers, the ratio of base fertilizer to top-dressing fertilizer was 7:3. Top-dressing fertilizer was applied in combination at the jointing stage.

**Figure 1:**
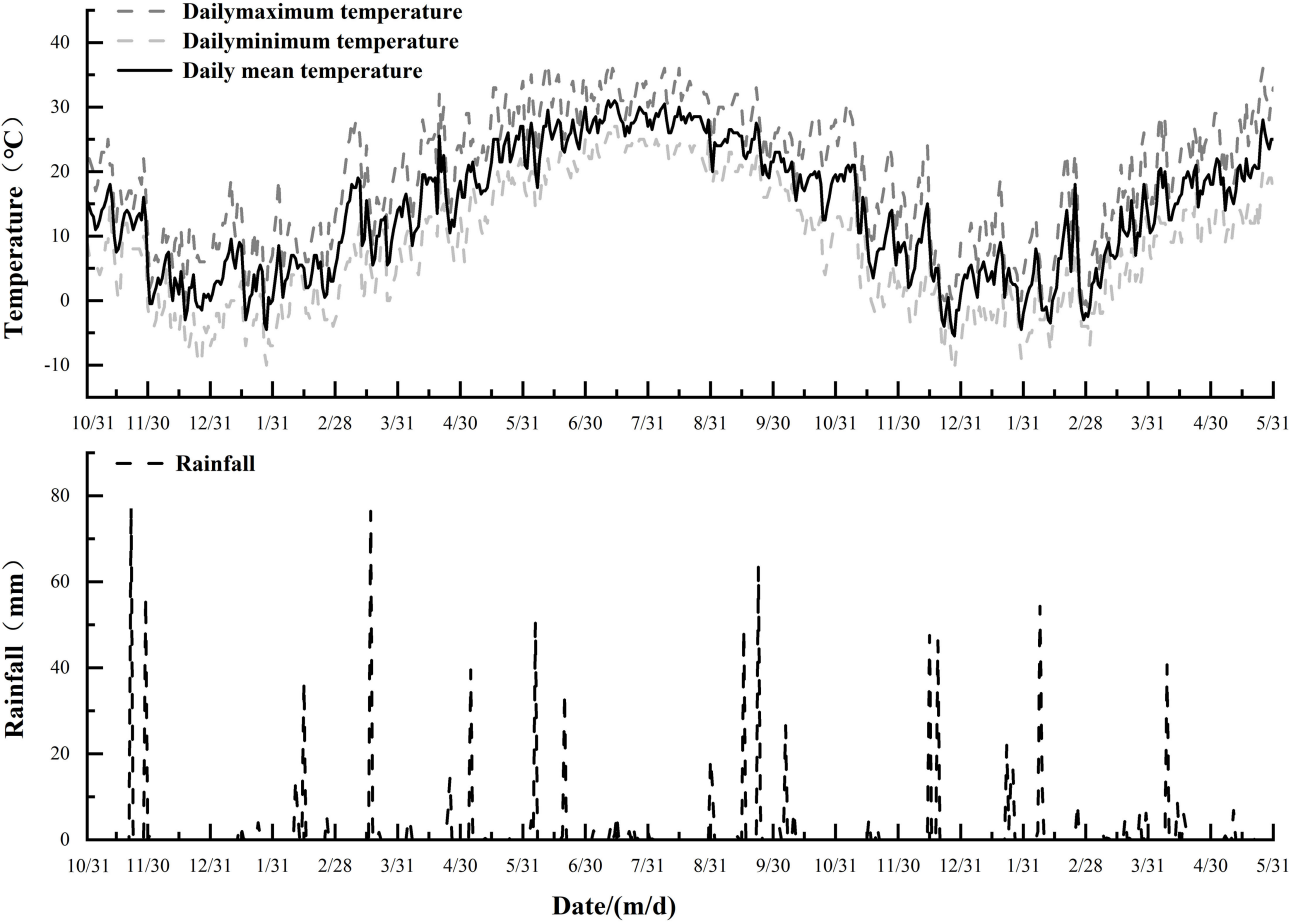
Meteorological data in wheat growth period from October 31, 2022 to May 31, 2024.

The experiment used a randomized block design and was conducted during 2022–2023 in the wheat filling stage (7–35 days post anthesis). Three shading levels were set: 10% shading (S1), 20% shading (S2) and 30% shading (S3), with natural light conditions used as the control (CK). In 2023–2024, two N application rates (120 and 180 kg/hm^2^, represented by N1 and N2, respectively) and the abovementioned three shading treatments for each N application rate were set during the wheat filling stage. The plot area was 9 m2 (3 m × 3 m), and the row spacing was 0.25 m, which was repeated three times. Grey nets with light transmission of approximately 90%, 80% and 70% were used to over the plants throughout the grain filling stage. The shading net was placed approximately 80 cm away from the surface of the wheat canopy to ensure good ventilation within the population for field observation and sampling. Wheat was sown on 31 October 2022 and harvested on 1 June 2023. Then, it was sown on 31 October 2023 and harvested on 1 June 2024. The field population microclimate data during the field experiments are presented in **Table 1**, and the temperature and precipitation data during the wheat growth period from 2022 to 2024 are shown in **Figure 1**. Other measures were identical to those in local high-yield fields.

**Table 1:** Effect of shading post anthesis on field microclimate.

Year	Cultivar	Treatment	Light intensity/ (μmol m-2 s-l)	CO2 concentration/ (μmol mol-l)	Air temperature/ (℃)	Humidity/ (%)
2022-2023	QM725	CK	1085.42 ± 42.16a	447.67 ± 1.53a	23.30 ± 0.56a	52.93 ± 0.90a
		S1	982.08 ± 4.39b	447.67 ± 2.52a	22.87 ± 0.60a	53.73 ± 0.55a
		S2	872.92 ± 4.02c	447.33 ± 4.04a	22.90 ± 0.61a	53.93 ± 0.87a
		S3	762.50 ± 20.12d	447.67 ± 2.08a	23.57 ± 0.06a	53.97 ± 0.64a
	YM15	CK	1116.25 ± 63.79a	445.67 ± 1.53a	23.10 ± 0.62a	53.77 ± 0.50a
		S1	1008.33 ± 15.93b	445.33 ± 1.53a	23.37 ± 0.15a	53.93 ± 0.99a
		S2	897.08 ± 7.11c	445.33 ± 1.53a	23.43 ± 0.06a	53.67 ± 0.25a
		S3	785.83 ± 7.11d	445.67 ± 1.53a	23.17 ± 0.25a	53.83 ± 0.55a
2023-2024	QM725	CK	1265.64 ± 549.05a	437.38 ± 4.36a	23.81 ± 3.59a	35.29 ± 2.78a
		S1	1116.67 ± 484.95b	439.27 ± 9.16a	24.53 ± 3.96a	35.79 ± 2.58a
		S2	977.78 ± 421.95c	439.37 ± 7.56a	24.23 ± 3.80a	35.71 ± 3.00a
		S3	836.01 ± 364.99d	438.89 ± 6.66a	24.36 ± 3.63a	36.09 ± 1.87a
	YM15	CK	1290.76 ± 571.37a	437.78 ± 7.98a	24.51 ± 4.22a	35.31 ± 5.12a
		S1	1138.23 ± 505.09b	439.12 ± 8.22a	24.75 ± 3.98a	35.62 ± 3.91a
		S2	1003.41 ± 443.68c	438.54 ± 7.74a	24.56 ± 3.83a	35.54 ± 2.92a
		S3	855.31 ± 376.60d	437.56 ± 3.57a	24.31 ± 3.71a	35.95 ± 3.61a

The data in the table are the average values of 7-35 days during the weak light test during the grouting period from 2022 to 2024. CK: No shading; S1: 10% shading; S2: 20% shading; S3: 30% shading. Different lowercase letters in the same column indicated significant difference among different treatments of the same variety (*P*< 0.05); The number after "±" represents the standard deviation. The following table is the same.

### 2.2 Measurement and methods

#### 2.2.1 Field microclimate

The Lx-101 luminance meter was used to measure light intensity. A portable CO_2_ analyzer was used to measure CO_2_ concentration. The DWHJ-2 temperature and humidity meter was used to measure the temperature and humidity. The data were measured from 7:00 to 19:00 each day for 35 days, and the average values were recorded.

#### 2.2.2 Yield and yields components

At the maturity stage of wheat grains, three 1m^2^ area regions were selected in each plot to measure the number of effective wheat spikes. Thirty wheat spikes were randomly selected and the number of grains per spike was counted. All wheat spikes in the sample area were harvested, dried, threshed and weighed to determine the actual yield. Finally, 1000 grains were randomly selected and weighed to determine the 1000-grain weight in triplicate.

#### 2.2.3 Starch and its component contents

The total starch and amylose contents of wheat grains were determined using the Megazyme International Ireland kit. The amylopectin content was determined by calculating the difference between the total starch content and amylose content.

#### 2.2.4 Starch particle size distribution

The starch content was improved and extracted according to the method of Peña-Bautista et al. (2017). The volume, surface area percentage and number of starch granules were determined using a laser diffraction particle size analyzer (LS13320, Beckman Coulter, USA).

#### 2.2.5 Gelatinization characteristics of starch

The wheat grains were ground into flour using Laboratory Mill 3100 (Perten Company). Briefly, 3 g of flour was placed in an aluminum box, and 25 mL of distilled water was added. The box was placed in a Starchmaster-2 rapid viscosity analyzer to measure the Rapid Visco-Analyzer parameters of the flour.

#### 2.2.6 Thermodynamic characteristics of starch

Based on the method of Kaczmarek et al. (2017), the thermodynamic characteristics of starch were determined using a DSC-3 (Switzerland-Mettler-Toledo) differential scanning calorimeter. Briefly, 3.0 mg of starch sample was weighed and mixed with 9 µL of deionized water. The samples were placed on an aluminum crucible, sealed and allowed to equilibrate at room temperature for 24 h before measurement. An empty crucible was used as the control. The samples were analyzed at the starting temperature of 20°C, scanning rate of 10°C/min and maximum temperature of 100°C. Heat change during scanning was measured.

### 2.3 Statistical analysis

Microsoft Excel 2021 software was used for data processing and tabulation. The LSD method in DPS 7.05 software was used for significance testing and variance analysis. Drawing was performed using Origin 2021.

## 3 Results

### 3.1 Yield and yield components

Compared with CK, weak light treatment post anthesis had no significant effect on the number of wheat spikes but exerted a significant effect on the grain number per spike, 1000-grain weight and grain yield; the effect was consistent over the 2 years (**Table 2**). In 2022, 2023 and 2024, following weak light treatment post anthesis, the number of grains per spike for the two wheat varieties (QM725 and YM15) decreased by 5.02%, 14.43% and 26.79% and 5.94%, 11.75% and 20.92%, respectively. The 1000-grain weight decreased by 11.68%, 20.67% and 29.42% and 7.91%, 16.14% and 26.29%, respectively. The grain yield decreased by 19.14%, 33.18% and 45.57% and 19.03%, 26.05% and 39.46%, respectively. With the enhancement of N application rates, the grain yield and yield components of wheat significantly improved; however, weak light treatment under the same N fertilizer condition significantly reduced these parameters. The same trend was observed in both varieties. These results revealed that weak light treatment post anthesis is not conducive to improving wheat yield and yield components. The decrease in 1000-grain weight was the most pronounced across all treatments, indicating that yield reduction is mainly mediated by altering1000-grain weight. In contrast, increasing N fertilizer application rates is considered beneficial in increasing grain weight and yield.

**Table 2:** Effects of nitrogen application rate and weak light post anthesis on wheat yield and its components.

Year	Cultivar	Treatment		Spike number/ (×10 ^4^·hm^-2^)	Grain number per spike	1000-grain weight / (g)	Yield / (kg·hm^-2^)
2022-2023	QM725	CK		634.67 ± 16.17	51.63 ± 0.15	35.49 ± 1.75	7997.87 ± 222.32
		S1		632.00 ± 34.87	51.10 ± 0.40	31.07 ± 1.54	6290.78 ± 222.13
		S2		629.33 ± 8.33	44.83 ± 0.55	29.60 ± 1.71	5074.87 ± 162.24
		S3		624.00 ± 18.33	40.00 ± 2.55	26.15 ± 1.42	3524.20 ± 61.03
	YM15	CK		648.00 ± 24.33	49.57 ± 0.85	38.20 ± 1.84	8360.93 ± 129.38
		S1		644.00 ± 6.93	47.90 ± 0.44	35.07 ± 1.19	6711.60 ± 142.71
		S2		638.67 ± 26.63	44.67 ± 0.81	30.96 ± 2.00	5842.47 ± 329.28
		S3		634.67 ± 12.22	43.37 ± 0.29	28.91 ± 0.71	4726.49 ± 204.27
	*F value*	Cultivar C		1.77	0.23	21.82**	64.79**
		Shading S		0.37	109.82**	47.20*	400.56**
		C×S		0.01	10.74**	0.86	5.10*
2023-2024	QM725	N1	CK	633.33 ± 10.07	47.18 ± 1.26	36.70 ± 1.56	7346.89 ± 321.87
			S1	609.33 ± 16.65	43.93 ± 3.80	32.98 ± 2.05	5708.22 ± 151.32
			S2	625.33 ± 8.33	39.38 ± 1.62	28.26 ± 1.15	4698.93 ± 192.49
			S3	632.00 ± 28.00	31.02 ± 2.17	24.25 ± 1.49	3752.67 ± 321.62
		N2	CK	634.67 ± 24.11	48.80 ± 2.18	39.74 ± 0.85	7832.71 ± 239.39
			S1	617.33 ± 27.23	45.32 ± 1.62	34.79 ± 1.47	6753.11 ± 218.86
			S2	620.00 ± 18.33	42.17 ± 1.57	30.55 ± 0.95	5721.11 ± 256.77
			S3	614.67 ± 22.03	37.29 ± 1.62	28.60 ± 1.27	4359.73 ± 377.15
	YM15	N1	CK	614.67 ± 34.02	43.89 ± 0.99	32.50 ± 1.53	7845.47 ± 110.94
			S1	602.67 ± 33.31	39.05 ± 1.62	29.19 ± 0.26	5926.58 ± 103.13
			S2	609.33 ± 36.30	37.29 ± 1.62	27.49 ± 1.00	5600.04 ± 200.90
			S3	594.67 ± 34.02	29.71 ± 2.65	23.61 ± 0.78	4687.91 ± 188.29
		N2	CK	645.33 ± 38.44	50.53 ± 1.57	35.48 ± 0.49	8189.29 ± 183.23
			S1	630.67 ± 22.03	48.80 ± 2.18	33.58 ± 1.10	7132.89 ± 228.53
			S2	616.00 ± 16.00	45.32 ± 1.62	30.50 ± 0.73	6598.71 ± 276.07
			S3	621.33 ± 29.48	41.47 ± 1.62	25.83 ± 1.43	5350.80 ± 192.42
	*F value*	Cultivar C		0.72	0.05	41.44**	41.57**
		Nitrogen N		1.66	112.15**	76.87**	102.71**
		Shading S		1.10	90.70**	173.12**	527.63**
		C×N		2.97	28.00**	0.16	0.08
		C×S		0.28	0.87	5.38**	9.95**
		N×S		0.31	3.37*	0.15	7.14**
		C×N×S		0.20	0.47	2.02	0.25

The data in the table are the average values processed during the 2022-2024 test period. CK: No shading; S1: 10% shading; S2: 20% shading; S3: 30% shading. C×N: cultivar×nitrogen; C×S: cultivar×shading; N×S: nitrogen×shading; C×N×S: cultivar×nitrogen×shading interaction effect. * and ** indicate significant difference at the probability level of 0.05 and 0.01, respectively; The number after "±" represents the standard deviation. The following table is the same.

### 3.2 Starch and its components

As shown in **Table 3**, compared with CK, weak light treatment post anthesis had a significant effect on the content of wheat starch and its components, and the effect was consistent over the 2 years. In 2022–2024, following weak light treatment post anthesis, the total starch content of the two wheat varieties (QM725 and YM15) decreased by 4.40%, 8.39% and 10.28% and 3.36%, 4.82% and 7.07%, respectively. The amylose content decreased by 7.45%, 14.83% and 18.23% and 6.76%, 9.20% and 14.43%, respectively. The amylopectin content decreased by 3.15%, 5.63% and 6.18% and 1.87%, 2.85% and 3.86%, respectively. The amylose/amylopectin ratio decreased by 4.15%, 9.90% and 14.81% and 5.32%, 7.61% and 10.64%, respectively. With the enhancement of N application rates, the content of wheat starch and its components significantly improved; however, weak light treatment under the same N fertilizer condition resulted in a significant decrease in the content of wheat starch and its components. The same trend was observed in both varieties. These results revealed that weak light treatment post anthesis is not conducive to improving the content of wheat starch and its components. The decrease in amylose content was the largest across all treatments, indicating that the starch content is mainly reduced by altering the amylose content, and an appropriate increase in the amount of N fertilizer can alleviate the effect of weak light stress on the content of wheat grain starch.

**Table 3:** Effects of nitrogen application rate and weak light post anthesis on starch and its components in wheat.

Year	Cultivar	Treatment		Total Starch/ (%)	Amylose/ (%)	Amylopectin/ (%)	Amylose/Amylopectin/ (%)
2022-2023	QM725	CK		66.63 ± 0.06	18.26 ± 0.19	48.37 ± 0.15	0.38 ± 0.01
		S1		65.93 ± 0.12	17.90 ± 0.33	48.04 ± 0.22	0.37 ± 0.01
		S2		63.70 ± 1.66	16.36 ± 1.33	47.34 ± 0.37	0.35 ± 0.03
		S3		63.03 ± 0.12	15.77 ± 1.06	47.26 ± 0.98	0.33 ± 0.03
	YM15	CK		67.70 ± 0.17	20.24 ± 0.17	47.46 ± 0.23	0.43 ± 0.01
		S1		66.57 ± 0.21	19.19 ± 0.31	47.38 ± 0.12	0.41 ± 0.01
		S2		66.50 ± 0.17	19.15 ± 0.29	47.35 ± 0.23	0.40 ± 0.01
		S3		64.87 ± 0.21	18.07 ± 0.58	46.80 ± 0.78	0.39 ± 0.02
	*F value*	Cultivar C		42.01**	62.59**	7.06*	56.19**
		Shading S		32.67**	14.50**	4.24*	7.85**
		C×S		3.79*	1.42	1.04	0.77
2023-2024	QM725	N1	CK	66.77 ± 0.64	19.07 ± 0.64	47.70 ± 0.27	0.40 ± 0.01
			S1	62.24 ± 1.25	16.78 ± 0.55	45.46 ± 0.76	0.37 ± 0.01
			S2	58.17 ± 0.35	14.92 ± 0.54	43.25 ± 0.74	0.35 ± 0.02
			S3	57.73 ± 1.84	14.53 ± 0.83	43.20 ± 1.03	0.34 ± 0.01
		N2	CK	65.93 ± 0.61	19.96 ± 0.63	45.97 ± 1.21	0.43 ± 0.03
			S1	62.39 ± 1.63	18.29 ± 0.29	44.10 ± 1.35	0.42 ± 0.01
			S2	60.73 ± 0.76	17.00 ± 0.18	43.73 ± 0.65	0.39 ± 0.01
			S3	58.08 ± 0.46	15.45 ± 0.29	42.63 ± 0.73	0.36 ± 0.01
	YM15	N1	CK	65.96 ± 1.19	19.93 ± 0.61	46.03 ± 0.58	0.43 ± 0.01
			S1	62.79 ± 0.98	18.39 ± 0.26	44.40 ± 1.23	0.41 ± 0.02
			S2	60.98 ± 0.96	17.50 ± 0.32	43.48 ± 0.86	0.40 ± 0.01
			S3	59.35 ± 1.38	16.52 ± 1.02	42.83 ± 0.54	0.39 ± 0.02
		N2	CK	67.97 ± 0.41	20.96 ± 0.83	47.01 ± 1.21	0.45 ± 0.03
			S1	65.53 ± 0.55	19.41 ± 0.54	46.12 ± 0.38	0.42 ± 0.01
			S2	64.47 ± 1.08	18.76 ± 0.29	45.71 ± 1.02	0.41 ± 0.01
			S3	63.20 ± 0.73	17.72 ± 0.54	45.48 ± 0.52	0.39 ± 0.01
	*F value*	Cultivar C		73.74**	100.19**	7.96**	51.53**
		Nitrogen N		45.39**	56.45**	6.07*	26.99**
		Shading S		133.67**	104.82**	38.63**	34.00**
		C×N		21.66**	0.45	36.51**	11.43**
		C×S		6.09**	3.34*	2.57	0.96
		N×S		3.76*	0.91	3.14*	0.34
		C×N×S		1.06	0.63	0.55	0.28

The data in the table are the average values processed during the 2022-2024 test period. CK: No shading; S1: 10% shading; S2: 20% shading; S3: 30% shading. C×N: cultivar×nitrogen; C×S: cultivar×shading; N×S: nitrogen×shading; C×N×S: cultivar×nitrogen×shading interaction effect. * and ** indicate significant difference at the probability level of 0.05 and 0.01, respectively; The number after "±" represents the standard deviation. The following table is the same.

### 3.3 Starch particle size distribution

#### 3.3.1 Volume distribution

As shown in **Table 4**, compared with CK, weak light treatment post anthesis had a significant effect on the volume distribution of wheat starch granules, and the effect was consistent over the 2 years. In 2022–2024, following weak light treatment post anthesis, the volume percentage of B-type starch granules with a size of ≤10 μm for the two wheat varieties (QM725 and YM15) decreased by 10.90%, 19.38% and 30.61% and 12.09%, 17.23% and 23.47%, respectively. The volume percentage of A-type starch granules with a size of >10 μm increased by 5.94%, 10.65% and 24.75% and 6.19%, 8.80% and 11.99%, respectively. With the enhancement of N application rates and a decrease in light intensity following anthesis, the volume percentage of B-type starch granules significantly reduced, whereas that of A-type starch granules increased. The performance of the two wheat varieties was consistent. For B-type starch granules, the effect of each treatment on the volume percentage of starch granules with a size of 0.1–2.8 μm was greater than that on the volume percentage of starch granules with a size of 2.8–10 μm. For A-type starch granules, the effect of each treatment on the volume percentage of starch granules with a particle size of >22 μm was greater than that on the volume percentage of starch granules with a particle size of 10–22 μm. These results revealed that weak light treatment post anthesis is not conducive to increasing the volume percentage of wheat B-type starch granules, indicating that weak light treatment mainly affects the formation of B-type starch granules, thereby relatively increasing the percentage of A-type starch granules. Increasing the amount of N fertilizer was beneficial in increasing the percentage of starch granule volume.

**Table 4:** Effects of nitrogen application rate and weak light post anthesis on volume distribution of starch granules in wheat.

Year	Cultivar	Treatment		Diameter of starch granule/ (%)					
				0.1–2.8 μm	2.8–10 μm	≤10 μm	>10 μm	10–22 μm	>22 μm
2022-2023	QM725	CK		10.32 ± 0.54	22.35 ± 0.52	32.67 ± 1.04	67.33 ± 1.04	37.13 ± 0.63	30.20 ± 0.43
	S1			9.00 ± 0.22	19.81 ± 0.57	28.81 ± 0.79	71.19 ± 0.79	38.68 ± 0.81	32.51 ± 0.40
	S2			8.03 ± 0.71	18.27 ± 0.45	26.30 ± 1.17	73.70 ± 1.17	40.30 ± 0.58	33.40 ± 0.61
	S3			6.28 ± 0.10	15.22 ± 0.14	21.50 ± 0.20	78.50 ± 0.20	42.15 ± 0.02	36.35 ± 0.22
	YM15	CK		10.49 ± 0.51	23.40 ± 0.17	33.89 ± 0.68	66.11 ± 0.68	36.29 ± 0.25	29.82 ± 0.74
		S1		9.13 ± 0.37	20.08 ± 0.43	29.21 ± 0.78	70.79 ± 0.78	38.65 ± 0.24	32.14 ± 0.60
		S2		8.97 ± 0.44	19.49 ± 0.41	28.46 ± 0.81	71.54 ± 0.81	39.14 ± 0.46	32.40 ± 0.51
		S3		7.72 ± 0.17	18.40 ± 0.06	26.12 ± 0.23	73.88 ± 0.23	40.11 ± 0.07	33.77 ± 0.16
	*F value*	Cultivar C		15.56**	90.35**	48.04**	48.02**	28.04**	32.08**
		Shading S		69.06**	281.94**	168.22**	168.18**	94.09**	117.91**
		C×S		3.49*	17.03**	9.14**	9.14**	4.68*	7.48**
2023-2024	QM725	N1	CK	11.04 ± 0.04	25.99 ± 0.12	37.03 ± 0.16	62.97 ± 0.16	30.88 ± 0.23	32.09 ± 0.09
			S1	9.73 ± 0.04	22.21 ± 0.38	31.94 ± 0.39	68.06 ± 0.39	32.76 ± 0.43	35.30 ± 0.49
			S2	8.25 ± 0.12	20.61 ± 0.11	28.86 ± 0.22	71.14 ± 0.22	34.27 ± 0.61	36.87 ± 0.51
			S3	7.01 ± 0.04	18.92 ± 0.62	25.93 ± 0.65	74.07 ± 0.65	35.30 ± 0.09	38.77 ± 0.66
		N2	CK	11.96 ± 0.02	24.15 ± 0.05	36.11 ± 0.04	63.89 ± 0.04	33.35 ± 0.04	30.54 ± 0.03
			S1	10.20 ± 0.21	23.34 ± 0.22	33.54 ± 0.12	66.46 ± 0.12	34.52 ± 0.20	31.94 ± 0.23
			S2	8.75 ± 0.02	21.27 ± 0.57	30.02 ± 0.58	69.98 ± 0.58	35.12 ± 0.57	34.86 ± 0.15
			S3	7.84 ± 0.06	18.28 ± 0.05	26.12 ± 0.03	73.88 ± 0.03	37.91 ± 0.08	35.97 ± 0.06
	YM15	N1	CK	11.25 ± 0.34	23.18 ± 0.13	34.43 ± 0.28	65.57 ± 0.28	35.93 ± 0.57	29.64 ± 0.44
			S1	9.46 ± 0.02	20.18 ± 0.10	29.64 ± 0.11	70.36 ± 0.11	37.98 ± 0.08	32.38 ± 0.08
			S2	8.70 ± 0.24	18.37 ± 0.95	27.07 ± 0.74	72.93 ± 0.74	39.78 ± 0.70	33.15 ± 0.06
			S3	7.65 ± 0.03	17.30 ± 0.61	24.94 ± 0.58	75.06 ± 0.58	40.41 ± 0.29	34.65 ± 0.29
		N2	CK	10.62 ± 0.16	22.20 ± 0.56	32.82 ± 0.57	67.18 ± 0.57	36.60 ± 0.52	30.58 ± 0.22
			S1	9.33 ± 0.15	20.68 ± 0.29	30.01 ± 0.40	69.99 ± 0.40	37.64 ± 0.25	32.35 ± 0.18
			S2	8.55 ± 0.40	19.58 ± 0.14	28.13 ± 0.30	71.87 ± 0.30	37.96 ± 0.08	33.91 ± 0.33
			S3	8.11 ± 0.05	18.17 ± 0.09	26.28 ± 0.10	73.72 ± 0.10	39.67 ± 0.22	34.05 ± 0.25
	*F value*	Cultivar C		8.13**	249.64**	299.08**	299.08**	1297.50**	559.98**
		Nitrogen N		32.42**	0.93	11.47**	11.47**	37.97**	170.63**
		Shading S		960.13**	414.05**	1141.30**	1141.30**	248.09**	732.24**
		C×N		64.25**	5.73*	0.89	0.89	125.80**	267.68**
		C×S		26.57**	8.80**	25.83**	25.83**	2.7	28.59**
		N×S		5.98**	20.32**	22.65**	22.65**	15.06 **	19.25**
		C×N×S		6.91**	3.53*	4.70**	4.70**	2.36	2.11

The data in the table are the average values processed during the 2022-2024 test period. CK: No shading; S1: 10% shading; S2: 20% shading; S3: 30% shading. C×N: cultivar×nitrogen; C×S: cultivar×shading; N×S: nitrogen×shading; C×N×S: cultivar×nitrogen×shading interaction effect. * and ** indicate significant difference at the probability level of 0.05 and 0.01, respectively; The number after "±" represents the standard deviation. The following table is the same.

#### 3.3.2 Surface area distribution

As shown in **Table 5**, compared with CK, weak light treatment post anthesis had a significant effect on the surface area distribution of wheat starch granules, and the effect was consistent over the 2 years. In 2022–2024, following weak light treatment post anthesis, the surface area percentage of B-type starch granules with a size of ≤10 μm for the two wheat varieties (QM725 and YM15) decreased by 5.24%, 8.70% and 13.94% and 5.63%, 9.13% and 13.02%, respectively. The surface area percentage of A-type starch granules with a size of >10 μm increased by 22.76%, 37.77% and 60.76% and 23.56%, 38.97% and 55.23%, respectively. With the enhancement of N application rates and a decrease in light intensity following anthesis, the surface area percentage of B-type starch granules significantly reduced, whereas that of A-type starch granules increased. The performance of the two wheat varieties was consistent. For B-type starch granules, the effect of each treatment on the surface area percentage of starch granules with a particle size of 0.1–2.8 μm was greater than that on the surface area percentage of starch granules with a particle size of 2.8–10 μm. For A-type starch granules, the effect of each treatment on the surface area percentage of starch granules with a particle size of >22 μm was greater than that on the surface area percentage of starch granules with a particle size of 10–22 μm. These results revealed that weak light treatment post anthesis is not conducive to increasing the percentage of surface area of wheat B-type starch granules, indicating that weak light treatment after anthesis weakens the synthesis of photosynthetic products. The amount of photosynthetic substances that can be differentiated into B-type starch granules is reduced, whereas that of photosynthetic substances that can be differentiated A-type starch granules is increased. An appropriate increase in the N application rate helps promote wheat photosynthesis.

**Table 5:** Effects of nitrogen application rate and weak light post anthesis on the surface area distribution of wheat starch granules.

Year	Cultivar	Treatment		Diameter of starch granule/ (%)					
				0.1–2.8 μm	2.8–10 μm	≤10 μm	>10 μm	10–22 μm	>22 μm
2022-2023	QM725	CK		49.43 ± 0.60	32.18 ± 0.49	81.61 ± 0.95	18.39 ± 0.95	11.20 ± 0.85	7.19 ± 0.57
		S1		46.26 ± 0.96	30.73 ± 0.39	76.99 ± 1.24	23.01 ± 1.24	14.48 ± 0.75	8.53 ± 0.59
		S2		44.90 ± 0.56	29.07 ± 0.66	73.97 ± 1.02	26.03 ± 1.02	16.23 ± 0.83	9.80 ± 1.28
		S3		40.58 ± 0.50	24.26 ± 0.11	64.84 ± 0.50	35.16 ± 0.50	20.58 ± 0.68	14.58 ± 0.24
	YM15	CK		48.09 ± 0.39	34.77 ± 0.38	82.87 ± 0.23	17.13 ± 0.23	10.77 ± 0.17	6.37 ± 0.28
		S1		47.55 ± 0.40	30.73 ± 0.24	78.29 ± 0.64	21.71 ± 0.64	13.51 ± 0.33	8.21 ± 0.44
		S2		44.68 ± 0.88	28.80 ± 0.44	73.48 ± 1.03	26.52 ± 1.03	16.43 ± 0.52	10.09 ± 0.53
		S3		43.35 ± 0.60	26.99 ± 0.86	70.34 ± 1.23	29.66 ± 1.23	17.81 ± 0.84	11.85 ± 0.41
	*F value*	Cultivar C		5.75*	34.82**	24.76**	24.76**	11.85**	16.47**
		Shading S		126.16**	236.00**	267.52**	267.50**	145.74**	155.60**
		C×S		11.85**	14.26**	11.25**	11.25**	4.89*	8.74**
2023-2024	QM725	N1	CK	52.87 ± 0.93	28.32 ± 0.53	81.19 ± 0.58	18.81 ± 0.58	13.27 ± 0.46	5.54 ± 0.25
			S1	50.15 ± 0.23	26.83 ± 0.20	76.98 ± 0.20	23.02 ± 0.20	15.85 ± 0.67	7.17 ± 0.50
			S2	47.54 ± 0.33	26.21 ± 0.11	73.75 ± 0.23	26.25 ± 0.23	18.40 ± 0.14	7.85 ± 0.09
			S3	46.56 ± 0.04	25.24 ± 0.59	71.80 ± 0.56	28.20 ± 0.56	19.29 ± 0.05	8.91 ± 0.55
		N2	CK	52.57 ± 0.48	28.41 ± 0.44	80.98 ± 0.60	19.02 ± 0.60	12.79 ± 0.60	6.23 ± 0.02
			S1	49.39 ± 0.62	27.64 ± 0.28	77.03 ± 0.55	22.97 ± 0.55	15.18 ± 0.35	7.79 ± 0.20
			S2	48.64 ± 0.14	26.21 ± 0.25	74.85 ± 0.13	25.15 ± 0.13	16.74 ± 0.07	8.41 ± 0.06
			S3	47.33 ± 0.74	25.82 ± 0.14	73.15 ± 0.66	26.85 ± 0.66	17.84 ± 0.64	9.01 ± 0.04
	YM15	N1	CK	53.31 ± 0.32	26.31 ± 0.32	79.62 ± 0.26	20.38 ± 0.26	13.25 ± 0.40	7.13 ± 0.14
			S1	50.07 ± 0.49	25.35 ± 0.09	75.42 ± 0.55	24.58 ± 0.55	15.61 ± 0.61	8.97 ± 0.56
			S2	49.46 ± 0.21	24.76 ± 0.58	74.22 ± 0.58	25.78 ± 0.58	16.04 ± 0.53	9.74 ± 0.67
			S3	47.87 ± 0.14	23.37 ± 0.09	71.24 ± 0.10	28.76 ± 0.10	18.19 ± 0.20	10.57 ± 0.10
		N2	CK	53.69 ± 0.14	25.64 ± 0.21	79.33 ± 0.09	20.67 ± 0.09	14.16 ± 0.02	6.51 ± 0.07
			S1	50.01 ± 0.08	24.50 ± 0.28	74.51 ± 0.29	25.49 ± 0.29	17.03 ± 0.42	8.46 ± 0.66
			S2	48.32 ± 0.27	23.65 ± 0.54	71.97 ± 0.32	28.03 ± 0.32	19.51 ± 0.19	8.52 ± 0.13
			S3	45.42 ± 0.21	23.28 ± 0.56	68.70 ± 0.37	31.30 ± 0.37	21.26 ± 0.60	10.04 ± 0.83
	*F value*	Cultivar C		10.39**	431.21 **	213.96**	213.96**	30.30**	88.70**
		Nitrogen N		6.63*	1.98	13.49**	13.49**	19.97**	0.90
		Shading S		492.78**	121.03**	939.56**	939.56**	373.04**	131.18**
		C×N		17.92**	23.76**	67.64**	67.64**	162.45**	25.66**
		C×S		4.65**	0.59	4.92**	4.92**	2.35	0.65
		N×S		2.89*	2.59	0.40	0.40	1.69	0.64
		C×N×S		17.41**	1.06	13.42**	13.42**	12.58**	1.00

The data in the table are the average values processed during the 2022-2024 test period. CK: No shading; S1: 10% shading; S2: 20% shading; S3: 30% shading. C×N: cultivar×nitrogen; C×S: cultivar×shading; N×S: nitrogen×shading; C×N×S: cultivar×nitrogen×shading interaction effect. * and ** indicate significant difference at the probability level of 0.05 and 0.01, respectively; The number after "±" represents the standard deviation. The following table is the same.

#### 3.3.3 Number distribution

As shown in **Table 6**, compared with CK, weak light treatment post anthesis had no significant effect on the number distribution of starch granules, whereas N application rate and weak light treatment post anthesis had significant effects on the number distribution of wheat starch granules. In 2022–2024, weak light treatment post anthesis significantly decreased the number percentage of starch granules with a size of 0.1–2.8 μm and significantly increased the number percentage of starch granules with a size of 2.8–10 μm. The number percentage of B-type starch granules accounted for 99.84%–99.87% of the total number percentage of all granules, indicating that most of the granules in the wheat grain analyzed were B-type starch granules. With the decrease in light intensity post anthesis, the number percentage of B-type starch granules significantly reduced, and the number percentage of A-type starch granules significantly increased. The performance of the two wheat varieties was consistent. N application and weak light treatment post anthesis resulted in a decrease in the percentage of B-type starch granules in wheat, indicating that the effect of N application and weak light treatment post anthesis was greater on B-type starch granules than on A-type starch granules.

**Table 6:** Effects of nitrogen application rate and weak light post anthesis on the number distribution of starch granules in wheat.

Year	Cultivar	Treatment		Diameter of starch granule/ (%)					
				0.1–2.8 μm	2.8–10 μm	≤10 μm	>10 μm	10–22 μm	>22 μm
2022-2023	QM725	CK		97.91 ± 0.06	1.98 ± 0.06	99.89 ± 0.01	0.11 ± 0.01	0.09 ± 0.01	0.02 ± 0.01
		S1		97.89 ± 0.05	1.99 ± 0.05	99.88 ± 0.01	0.12 ± 0.01	0.10 ± 0.01	0.02 ± 0.01
		S2		97.89 ± 0.59	1.99 ± 0.60	99.88 ± 0.01	0.12 ± 0.01	0.10 ± 0.00	0.02 ± 0.01
		S3		97.49 ± 0.03	2.37 ± 0.03	99.86 ± 0.03	0.14 ± 0.03	0.12 ± 0.03	0.02 ± 0.00
	YM15	CK		97.89 ± 0.06	2.00 ± 0.11	99.88 ± 0.07	0.12 ± 0.07	0.09 ± 0.05	0.03 ± 0.02
		S1		97.86 ± 0.08	2.01 ± 0.08	99.87 ± 0.01	0.13 ± 0.01	0.11 ± 0.01	0.02 ± 0.01
		S2		97.69 ± 0.07	2.19 ± 0.07	99.88 ± 0.00	0.12 ± 0.00	0.09 ± 0.01	0.03 ± 0.01
		S3		97.65 ± 0.49	2.22 ± 0.45	99.87 ± 0.04	0.13 ± 0.04	0.10 ± 0.04	0.03 ± 0.01
	*F value*	Cultivar C		0.04	0.03	0.10	0.07	0.22	3.87
		Shading S		1.65	1.55	0.72	0.46	0.74	0.26
		C×S		0.41	0.40	0.28	0.18	0.27	0.05
2023-2024	QM725	N1	CK	97.78 ± 0.04	2.10 ± 0.04	99.88 ± 0.01	0.12 ± 0.01	0.10 ± 0.00	0.02 ± 0.01
			S1	97.70 ± 0.08	2.16 ± 0.09	99.86 ± 0.01	0.14 ± 0.01	0.11 ± 0.00	0.03 ± 0.01
			S2	97.65 ± 0.04	2.22 ± 0.04	99.87 ± 0.00	0.13 ± 0.00	0.10 ± 0.00	0.03 ± 0.00
			S3	97.50 ± 0.05	2.35 ± 0.05	99.85 ± 0.00	0.15 ± 0.00	0.13 ± 0.00	0.02 ± 0.00
		N2	CK	97.73 ± 0.04	2.13 ± 0.04	99.86 ± 0.00	0.14 ± 0.00	0.12 ± 0.00	0.02 ± 0.00
			S1	97.69 ± 0.07	2.17 ± 0.07	99.86 ± 0.01	0.14 ± 0.01	0.12 ± 0.01	0.02 ± 0.00
			S2	97.68 ± 0.04	2.18 ± 0.04	99.86 ± 0.00	0.14 ± 0.00	0.12 ± 0.00	0.02 ± 0.00
			S3	97.61 ± 0.07	2.23 ± 0.08	99.84 ± 0.01	0.16 ± 0.01	0.13 ± 0.01	0.03 ± 0.00
	YM15	N1	CK	97.62 ± 0.09	2.25 ± 0.08	99.87 ± 0.01	0.13 ± 0.01	0.11 ± 0.01	0.02 ± 0.01
			S1	97.58 ± 0.02	2.28 ± 0.02	99.86 ± 0.01	0.14 ± 0.01	0.12 ± 0.01	0.02 ± 0.00
			S2	97.48 ± 0.06	2.38 ± 0.05	99.86 ± 0.01	0.14 ± 0.01	0.12 ± 0.01	0.02 ± 0.01
			S3	97.43 ± 0.02	2.43 ± 0.02	99.86 ± 0.01	0.14 ± 0.01	0.11 ± 0.01	0.03 ± 0.00
		N2	CK	97.51 ± 0.04	2.34 ± 0.04	99.85 ± 0.00	0.15 ± 0.00	0.13 ± 0.00	0.02 ± 0.00
			S1	97.49 ± 0.06	2.37 ± 0.06	99.86 ± 0.00	0.14 ± 0.00	0.12 ± 0.00	0.02 ± 0.00
			S2	97.40 ± 0.07	2.45 ± 0.07	99.85 ± 0.01	0.15 ± 0.01	0.12 ± 0.00	0.03 ± 0.01
			S3	97.29 ± 0.10	2.54 ± 0.10	99.83 ± 0.01	0.17 ± 0.01	0.14 ± 0.01	0.03 ± 0.00
	*F value*	Cultivar C		120.84**	120.22**	13.36**	13.36**	23.26**	0.19
		Nitrogen N		5.70*	3.15**	53.45**	53.45**	132.26**	1.71
		Shading S		24.48**	21.08**	33.73**	33.73**	37.21**	8.80**
		C×N		11.75**	11.09**	4.36*	4.36*	3.44	1.71
		C×S		0.66	0.58	1.00	1.00	3.08*	3.23*
		N×S		0.69	0.76	6.55*	6.55*	9.32**	4.75**
		C×N×S		1.32	1.09	3.27*	3.27*	22.17**	6.77**

The data in the table are the average values processed during the 2022-2024 test period. CK: No shading; S1: 10% shading; S2: 20% shading; S3: 30% shading. C×N: cultivar×nitrogen; C×S: cultivar×shading; N×S: nitrogen×shading; C×N×S: cultivar×nitrogen×shading interaction effect. * and ** indicate significant difference at the probability level of 0.05 and 0.01, respectively; The number after "±" represents the standard deviation. The following table is the same.

### 3.4 Gelatinization characteristics of starch

As shown in **Table 7**, compared with CK, weak light treatment post anthesis had a significant effect on the gelatinization characteristics of wheat starch, and the effect was consistent over the 2 years. In 2022–2024, following weak light treatment post anthesis, the peak viscosity of the two wheat varieties (QM725 and YM15) decreased by 15.50%, 25.20% and 38.28% and 18.28%, 30.09% and 37.52%, respectively. The trough viscosity decreased by 16.01%, 24.88% and 35.71% and 18.83%, 27.36% and 38.38%, respectively. The final viscosity decreased by 11.73%, 19.59% and 32.41% and 15.87%, 26.05% and 37.03%, respectively. The breakdown values decreased by 14.33%, 24.66% and 43.12%, and 17.06%, 34.86% and 43.09%, respectively. The setback values decreased by 8.09%, 14.62% and 29.57% and 12.76%, 19.52% and 41.98%, respectively. With the enhancement of N application rates, the gelatinization characteristics of wheat starch significantly improved; however, weak light treatment under the same N fertilizer conditions significantly reduced the gelatinization characteristics of wheat starch, with the two wheat varieties exhibiting the same trend. These results revealed that weak light treatment post anthesis is not conducive to improving the gelatinization characteristics of wheat starch, such as peak viscosity, trough viscosity and final viscosity. The decrease in N1 application rate was the largest across all treatments, indicating that the gelatinization characteristics of wheat starch are more susceptible to weak light stress in the absence of N fertilizer, and increasing the amount of N fertilizer can alleviate weak light stress.

**Table 7:** Effects of nitrogen application rate and weak light post anthesis on gelatinization characteristics of wheat starch.

Year	Cultivar	Treatment		Gelatinisation characteristics/ (cP)					
				Peak viscosity		Trough viscosity	Final viscosity	Breakdown	Setback
2022-2023	QM725	CK		1354.00 ± 54.81		915.00 ± 22.72	1695.67 ± 13.87	439.00 ± 34.18	780.67 ± 14.01
		S1		1174.33 ± 6.11		804.67 ± 22.28	1559.00 ± 33.60	369.67 ± 17.79	754.33 ± 18.34
		S2		1056.33 ± 26.39		722.33 ± 20.60	1460.67 ± 17.79	334.00 ± 13.45	738.33 ± 3.21
		S3		914.00 ± 12.12		644.00 ± 9.85	1295.67 ± 12.50	270.00 ± 5.00	651.67 ± 7.77
	YM15	CK		1526.33 ± 34.24		981.67 ± 14.50	1729.67 ± 27.74	544.67 ± 19.86	748.00 ± 38.97
		S1		1319.67 ± 32.47		892.00 ± 36.00	1551.33 ± 17.62	427.67 ± 15.04	659.33 ± 26.58
		S2		1065.00 ± 14.80		734.33 ± 11.02	1316.67 ± 14.01	330.67 ± 24.38	582.33 ± 12.90
		S3		839.00 ± 45.51		615.33 ± 8.50	1091.00 ± 85.46	223.67 ± 38.55	475.67 ± 77.36
	*F value*	Cultivar C		30.28**		22.07**	45.92**	12.60**	72.34**
		Shading S		450.56**		361.78**	351.36**	166.67**	39.44**
		C×S		26.03**		12.98**	22.31**	17.38**	5.74**
2023-2024	QM725	N1	CK	1315.33 ± 46.14		896.67 ± 50.72	1648.67 ± 22.19	418.67 ± 12.50	752.00 ± 40.00
			S1	1030.67 ± 22.68		665.33 ± 25.58	1338.33 ± 41.02	365.33 ± 10.07	673.00 ± 19.97
			S2	885.00 ± 29.31		575.67 ± 16.50	1209.67 ± 30.66	309.33 ± 24.58	634.00 ± 16.09
			S3	698.00 ± 21.28		466.33 ± 32.35	994.67 ± 38.07	231.67 ± 14.01	528.33 ± 20.01
		N2	CK	1416.33 ± 9.29		907.00 ± 32.51	1717.67 ± 55.47	509.33 ± 25.79	810.67 ± 41.65
			S1	1250.33 ± 24.58		814.67 ± 26.10	1540.67 ± 75.00	435.67 ± 18.72	726.00 ± 50.48
			S2	1133.00 ± 32.79		745.67 ± 20.40	1371.67 ± 42.67	387.33 ± 27.32	626.00 ± 23.81
			S3	913.33 ± 32.56		639.33 ± 14.57	1105.67 ± 38.42	274.00 ± 18.33	466.33 ± 43.78
	YM15	N1	CK	1347.67 ± 36.50		860.00 ± 33.15	1783.00 ± 28.16	487.67 ± 10.02	923.00 ± 52.26
			S1	1057.33 ± 33.13		658.00 ± 30.27	1468.00 ± 24.27	399.33 ± 7.02	810.00 ± 43.21
			S2	940.00 ± 13.86		640.00 ± 8.72	1283.00 ± 46.60	300.00 ± 20.30	643.00 ± 38.00
			S3	882.67 ± 21.55		525.67 ± 13.87	1008.67 ± 25.01	357.00 ± 14.80	483.00 ± 19.31
		N2	CK	1345.67 ± 7.37		897.00 ± 19.67	1596.33 ± 26.54	448.67 ± 23.18	699.33 ± 46.18
			S1	1079.67 ± 30.04		683.00 ± 22.61	1283.00 ± 57.47	396.67 ± 10.02	600.00 ± 41.80
			S2	944.67 ± 9.07		616.33 ± 22.90	1177.67 ± 15.04	328.33 ± 30.66	561.33 ± 29.40
			S3	801.00 ± 32.92		547.67 ± 29.87	954.33 ± 21.36	253.33 ± 6.66	406.67 ± 22.03
	*F value*	Cultivar C		13.91**		21.53**	15.46**	1.03	1.18
		Nitrogen N		124.29**		85.23**	0.02	17.66**	44.00**
		Shading S		764.72**		363.23**	559.16**	277.19**	177.57**
		C×N		166.05**		52.62**	128.54**	103.78**	58.34**
		C×S		9.53**		2.42	2.25	13.37**	3.06*
		N×S		5.58**		4.60**	3.04*	13.57**	0.66
		C×N×S		6.36**		9.88**	3.69*	5.46**	10.52**

The data in the table are the average values processed during the 2022-2024 test period. CK: No shading; S1: 10% shading; S2: 20% shading; S3: 30% shading. C×N: cultivar×nitrogen; C×S: cultivar×shading; N×S: nitrogen×shading; C×N×S: cultivar×nitrogen×shading interaction effect. * and ** indicate significant difference at the probability level of 0.05 and 0.01, respectively; The number after "±" represents the standard deviation. The following table is the same.

### 3.5 Thermodynamic characteristics of starch

As shown in **Table 8**, compared with CK, weak light treatment post anthesis had a significant effect on the thermodynamic characteristics of wheat starch, and the performance was consistent over the 2 years. In 2022–2024, following weak light treatment post anthesis, the onset temperatures of the two wheat varieties (QM725 and YM15) decreased by 2.83%, 4.70% and 8.37% and 2.46%, 5.18% and 10.56%, respectively. The peak temperatures decreased by 2.80%, 5.36% and 7.16% and 4.13%, 7.39% and 11.17%, respectively. The end temperatures decreased by 3.76%, 4.96% and 7.31% and 3.43%, 7.01% and 11.02%, respectively. The enthalpy values increased by 14.71%, 19.40% and 43.50% and 21.37%, 44.38% and 54.59%, respectively. With the enhancement of N application rates, the thermodynamic characteristics of wheat starch significantly improved; however, weak light treatment under the same N fertilizer conditions significantly reduced the onset temperature, peak temperature and end temperature of wheat starch, with the two wheat varieties exhibiting the same trend. These results revealed that weak light treatment post anthesis is not conducive to improving the thermodynamic characteristics of wheat starch. Although the enthalpy parameters of starch increased, the onset temperature, peak temperature and end temperature reduced significantly. N2 treatment helps improve the thermodynamic characteristics of starch.

**Table 8:** Effects of nitrogen application rate and weak light post anthesis on thermodynamic characteristics of wheat starch.

Year	Cultivar	Treatment		Starch thermodynamic characteristics			
				Onset temperature/ (℃)	Peak temperature/ (℃)	End temperature/ (℃)	Gel enthalpy/ (J·g-1)
2022-2023	QM725	CK		64.80 ± 0.60	69.82 ± 0.23	70.68 ± 0.03	6.14 ± 0.09
		S1		63.65 ± 0.38	67.71 ± 0.22	68.84 ± 0.20	6.87 ± 0.44
		S2		63.34 ± 0.23	67.17 ± 0.24	67.77 ± 0.30	7.11 ± 0.36
		S3		60.32 ± 0.25	65.92 ± 0.51	66.20 ± 0.14	8.07 ± 0.19
	YM15	CK		66.32 ± 0.43	72.87 ± 0.11	73.59 ± 0.48	6.77 ± 0.47
		S1		65.43 ± 0.27	68.72 ± 0.67	70.65 ± 0.17	7.81 ± 0.45
		S2		64.26 ± 0.28	67.14 ± 0.67	68.13 ± 0.48	9.18 ± 0.05
		S3		61.32 ± 0.63	63.64 ± 0.58	65.61 ± 0.76	9.85 ± 0.10
	*F value*	Cultivar C		82.71**	5.87*	75.20**	97.12**
		Shading S		202.10**	226.87**	419.56**	60.81**
		C×S		2.02	37.45**	35.60**	6.13
2023-2024	QM725	N1	CK	65.68 ± 0.05	69.75 ± 0.34	73.58 ± 0.56	4.92 ± 0.51
			S1	63.03 ± 0.74	67.29 ± 0.22	69.42 ± 0.20	5.78 ± 0.13
			S2	61.88 ± 0.23	64.76 ± 0.24	68.34 ± 0.31	6.16 ± 0.21
			S3	58.84 ± 0.25	63.50 ± 0.53	66.75 ± 0.14	7.91 ± 0.43
		N2	CK	67.01 ± 0.06	71.13 ± 0.33	72.33 ± 0.58	6.16 ± 0.04
			S1	65.20 ± 0.73	69.82 ± 0.67	70.13 ± 0.40	7.07 ± 0.48
			S2	62.94 ± 0.44	67.48 ± 0.33	69.70 ± 0.70	7.22 ± 0.37
			S3	61.78 ± 0.56	66.20 ± 0.12	67.77 ± 0.47	8.52 ± 0.36
	YM15	N1	CK	68.52 ± 0.18	71.50 ± 0.11	74.53 ± 0.11	5.52 ± 0.04
			S1	66.63 ± 0.67	69.29 ± 0.67	71.89 ± 0.45	6.79 ± 0.38
			S2	64.80 ± 0.28	66.73 ± 0.49	68.03 ± 0.17	8.18 ± 0.33
			S3	60.18 ± 0.11	63.61 ± 0.10	65.50 ± 0.90	9.17 ± 0.39
		N2	CK	66.61 ± 0.99	71.97 ± 0.11	73.66 ± 0.64	6.02 ± 0.35
			S1	64.42 ± 0.82	69.37 ± 0.94	71.64 ± 0.17	7.57 ± 0.44
			S2	61.94 ± 0.29	66.48 ± 0.51	70.06 ± 0.51	8.99 ± 0.54
			S3	58.64 ± 0.13	64.92 ± 0.09	66.22 ± 0.36	9.16 ± 0.20
	*F value*	Cultivar C		20.74**	15.32**	11.74**	91.96**
		Nitrogen N		0.74**	117.17**	11.26**	62.06**
		Shading S		414.60**	515.75**	514.47**	165.83**
		C×N		183.11**	58.62**	0.05	7.03*
		C×S		12.94	9.82**	32.26**	12.17**
		N×S		5.00**	3.30*	20.18**	2.77
		C×N×S		0.91	3.49*	1.99	0.27

The data in the table are the average values processed during the 2022-2024 test period. CK: No shading; S1: 10% shading; S2: 20% shading; S3: 30% shading. C×N: cultivar×nitrogen; C×S: cultivar×shading; N×S: nitrogen×shading; C×N×S: cultivar×nitrogen×shading interaction effect. * and ** indicate significant difference at the probability level of 0.05 and 0.01, respectively; The number after "±" represents the standard deviation. The following table is the same.

### 3.6 Correlation analysis

**Figure 2:**
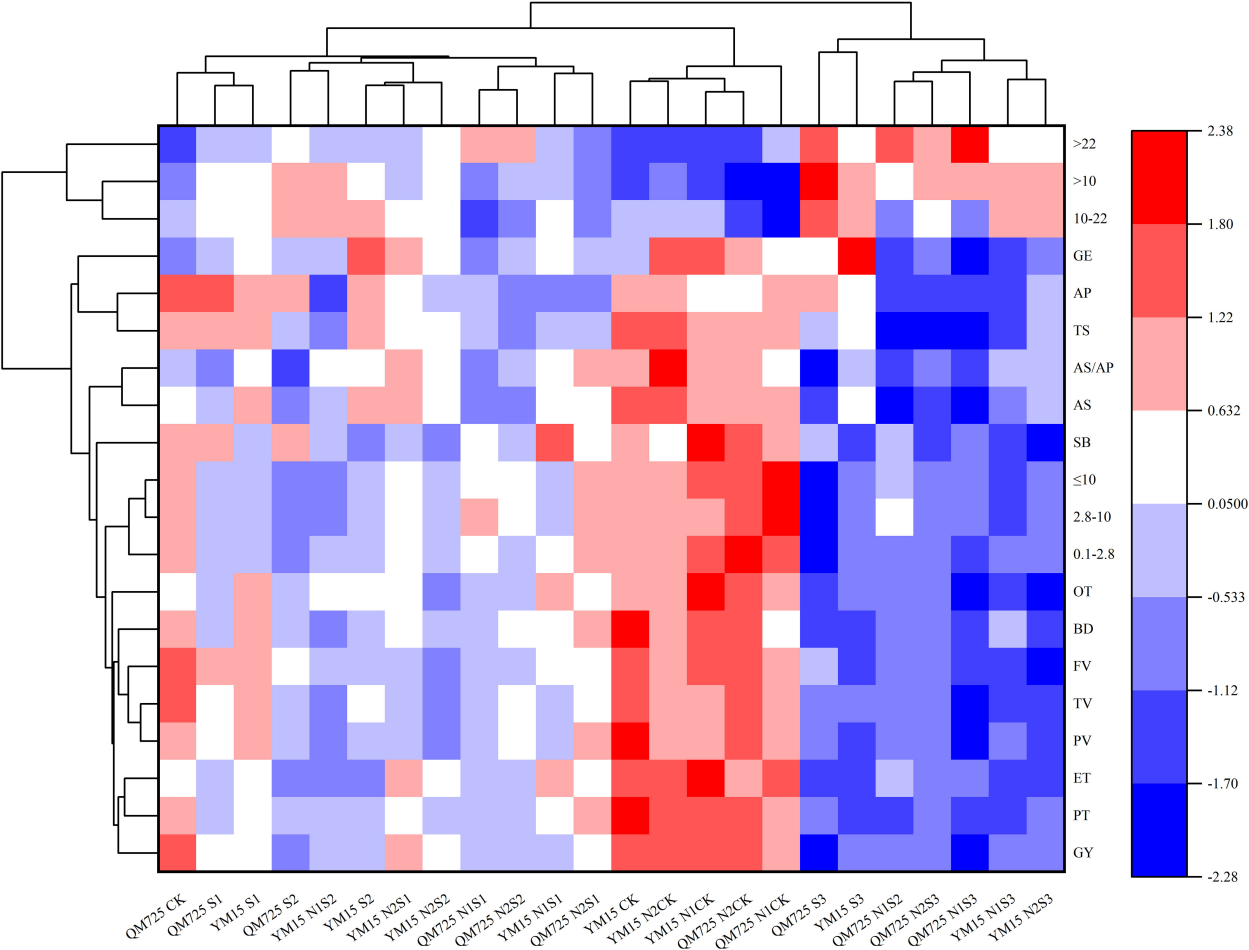
Correlation analysis between grain yield and physicochemical properties of wheat starch GY, Grain yield; TS, Total starch; AS, Amylose; AP, Amylopectin; AS/AP, Amylose/Amylopectin; PV, Peak viscosity; TV, Trough viscosity; FV, Final viscosity; BD, Breakdown; SB, Setback; OT, Onset temperature; PT, Peak temperature; ET, End temperature; GE, Gel enthalpy. The same figure below.

As shown in **Figure 2**, the grain yield of soft wheat was significantly positively correlated with the contents of starch and its components, B-type starch granules with a volume distribution of ≤10 μm, gelatinization characteristics of starch and thermodynamic characteristics of starch. In contrast, it was significantly negatively correlated with A-type starch granules with a volume distribution of >10 μm, contents of starch and its components. B-type starch granules with a volume distribution of ≤10 μm, gelatinization characteristics of starch and thermodynamic characteristics of starch. The contents of starch and its components were significantly positively correlated with the gelatinization and thermodynamic characteristics of starch. Starch gelatinization characteristics and thermodynamic characteristics were significantly positively correlated.

### 3.7 Cluster analysis

**Figure 3:**
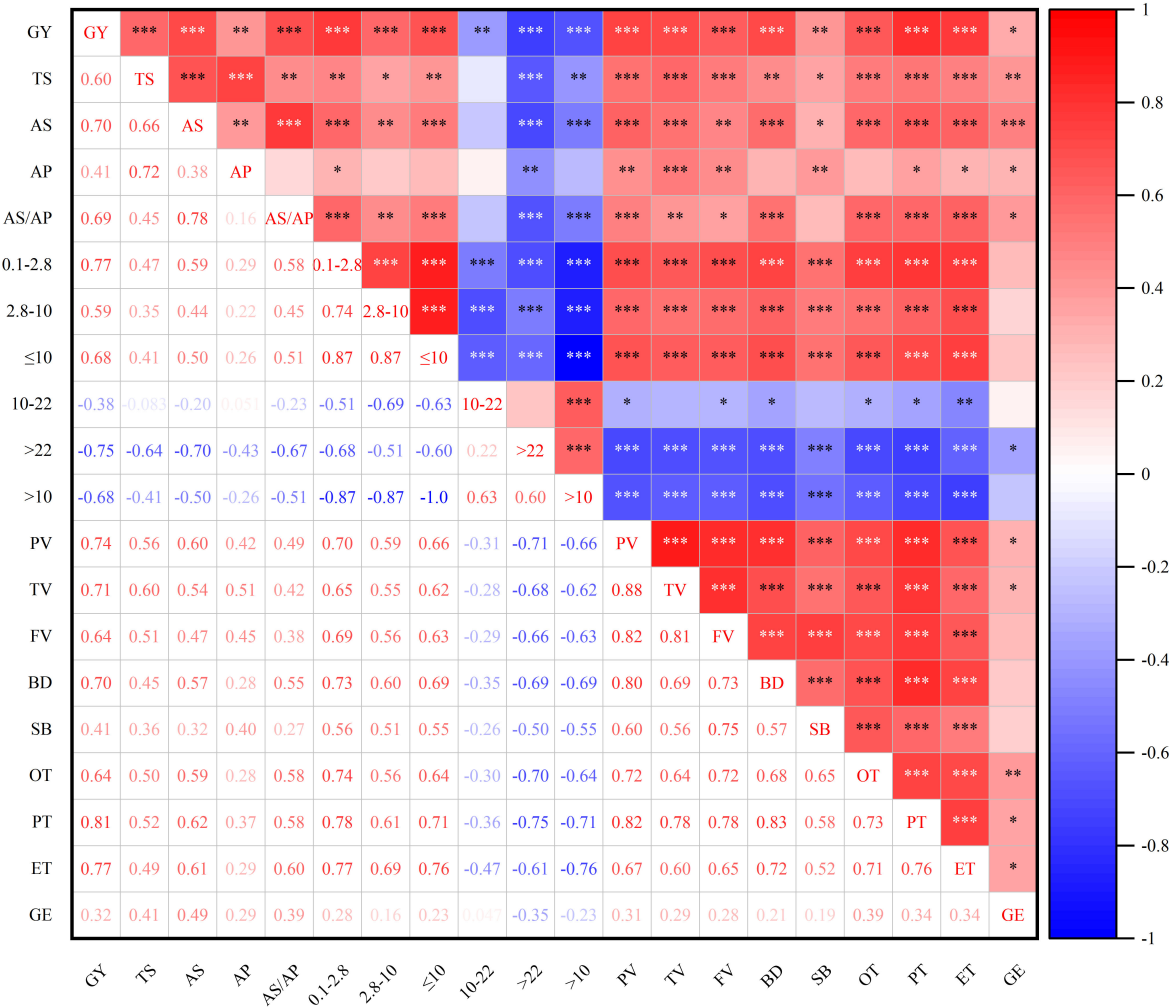
Cluster analysis of wheat grain yield and starch physicochemical properties. *, representing that p is less than 0.05, indicating that there is a significant difference between the data; ** represents that p is less than 0.01, indicating that there is a very significant difference between the data; ***, representing p<0.001, indicating that there is a very significant difference between the data.

The physical and chemical properties of wheat and starch were analyzed via cluster analysis. As shown in **Figure 3**, the 24 treatments were divided into 2 categories: class I (CK, S1 and S2) and class II (S3). The 20 indexes were divided into 2 categories: category I (A-type starch granules with a volume distribution of >10 μm) and category II (grain yield, contents of starch and its components, B-type starch granules with a volume distribution of ≤10 μm, starch gelatinization characteristics and starch thermodynamic characteristics). Cluster analysis revealed that the differences between the treatments were clear and the two categories may be divided into three subcategories. Of these, the S1 and S2 treatments were relatively close, whereas CK and S3 were quite different. The two categories of physicochemical properties of starch were also divided into three subcategories. Of these, A-type starch granules with a volume distribution of >10 μm were significantly different from other starch physicochemical properties. In contrast, B-type starch granules with a volume distribution of ≤10 μm were close to grain yield, contents of starch and its components, starch gelatinization characteristics and starch thermodynamic characteristics.

## 4 Discussion

Light is an important factor affecting grain yield and quality. The rate of N application also affects crop yield (Pagnani et al., 2024). Yang et al. (2019) showed that an increase in shading intensity had no significant effect on the number of spikes; however, the yield was reduced by decreasing the 1000-grain weight. Zhang et al. (2020) showed that top-dressing at the jointing stage may significantly increase the number of spikes and grains per spike, thereby increasing the crop yield. Wang et al. (2001) found that with an increase in N application rate, the number of spikes per unit area increased and the 1000-grain weight decreased. The present study showed that with an enhancement of N application rates, the number of grains per spike, 1000-grain weight and grain yield of wheat significantly increased. Under the same N application rate, the decrease in weak light treatment significantly reduced the number of grains per spike, 1000-grain weight and grain yield, whereas the effect on the number of wheat spikes did not reach a statistically significant level. Each treatment mainly reduced the yield by altering the 1000-grain weight of wheat, and appropriately increasing the amount of N fertilizer helped increase the yield of wheat. The effects of different weak light treatments on the 1000-grain weight and grain yield of QM725 were greater than those on the 1000-grain weight and grain yield of YM15. This indicates that the grain weight and yield of QM725 are more sensitive to weak light treatment than those of YM15, and the greater the shading intensity, the more significant the effect on grain weight and yield.

Wheat starch accounts for approximately 75% of the total grain dry weight (Shokat et al., 2024). It consists of amylose and amylopectin; amylose accounts for approximately one-third and amylopectin accounts for approximately two-third of the wheat starch (Labuschagne et al., 2007). The starch content is greatly affected by external environmental conditions; moreover, the quality traits of different wheat varieties differ due to environmental changes (Jin et al., 2019). Mou et al. (2009) revealed that the shading period from jointing to maturity significantly decreased the total starch, amylopectin and amylopectin/amylose contents of wheat but had no effect on the amylose content. Cai et al. (2007) demonstrated that with an increase in N application rates, the amylopectin content of wheat significantly increased, the amylose content and the ratio of amylose to amylopectin decreased and the accumulation of starch in wheat grains enhanced. Zhao et al. (2023) reported that different shading periods reduced the total starch, amylose and amylopectin contents in wheat grains; however, the amylose/amylopectin ratio showed only a slight change. The present study showed that the contents of starch and its components in wheat grains increased significantly with the increase in N application rates, but the contents of starch and its components in wheat grains decreased significantly. All weak light treatments under the same N application rate mainly reduced the total starch content by affecting the amylose content of wheat. An appropriate increase in the amount of N fertilizer was beneficial in increasing the starch content. The effect of different weak light treatments was greater on the starch content of QM725 than on the starch content of YM15, indicating that QM725 was more susceptible to weak light stress.

Starch particle size distribution is an important factor that affects wheat quality traits. In the wheat endosperm, starch exists in the form of starch granules, which are divided into A-type (≥10 μm) and B-type (<10 μm). The former accounts for 70% of the total starch weight, although the number distribution is <10%. The latter accounts for 30% of the total starch weight, but the number distribution accounts for >90% (Moon and Kweon, 2024; Studnicki et al., 2016). Yuan et al. (2020) demonstrated that weak light treatment during the filling stage reduced the percentage of B-type starch granules and increased the percentage of A-type starch granules. Ma et al. (2004) showed that increasing the rate of N fertilizer application could promote the growth of wheat A-type starch granules; increase their number and surface area percentage and reduce the number, volume and surface area percentage of B-type starch granules. The present study showed that with the enhancement of N application rate, the volume, surface area and number percentage of A-type and B-type starch granules significantly increased. Under the same N application rate, the decrease in weak light treatment significantly reduced the volume, surface area and number percentage of B-type starch granules and increased the volume, surface area and number percentage of A-type starch granules. The effects of different weak light treatments on the volume, surface area and number percentage of starch granules with particle size of 0.1–2.8 μm in the two wheat varieties were greater than those on the volume, surface area and number percentage of starch granules with particle size of 2.8–10 μm. For A-type starch granules, the effect of each treatment on the volume, surface area and number percentage of starch granules with particle size of >22 μm were greater than that on the volume, surface area and number percentage of starch granules with particle size of 10–22μm, indicating that the effect of weak light treatment post anthesis on the production and growth of wheat was greater on B-type starch granules than on A-type starch granules.

Starch gelatinization characteristics are important indicators of starch quality and particularly impact the edible quality of wheat (Watanabe et al., 2024). The thermodynamic characteristics of starch reflect its state and tend to change when it is heated in water. Enthalpy, an important thermodynamic characteristic of starch, represents the energy consumed by starch melting to make the starch semi-crystalline and soluble in water (Chatakanonda et al., 2000). Mou (2011) showed that shading reduced the peak viscosity and trough viscosity of wheat starch and increased the rebound value and gelatinization temperature. Gu et al. (2010) found that increasing the amount of N fertilizer had a significant effect on the gelatinization characteristics of wheat starch, but the effect depended on the genotype and amount of fertilizer. Liu et al. (2017) demonstrated that the content of grain starch and its components affected the thermodynamic characteristics of starch; they observed a decrease in the content of grain starch and its components due to insufficient light, which resulted in the alteration of the thermodynamic characteristics of starch. Liu et al. (2023) showed that the changes in the thermodynamic characteristics of different varieties of wheat starch may be related to the temperature used for the starch heating process in water. The present study showed that with the enhancement of N application rate, the gelatinization and thermodynamic characteristics of wheat starch significantly improved. Under the same N application rate, the weak light treatment significantly reduced the gelatinization and thermodynamic characteristics of wheat starch. Compared with N1, N2 was beneficial in improving the gelatinization and thermodynamic characteristics of wheat starch, indicating that the increase in N application rates was beneficial in improving the quality of wheat grains. Although the enthalpy parameters of starch increased, the onset temperature, peak temperature and end temperature significantly reduced, and the effect on the gelatinization characteristics, onset temperature, peak temperature and end temperature was less for QM725 than for YM15, indicating that YM15 was more susceptible to gelatinization temperature in this experiment.

Grain yield is closely related to the physicochemical characteristics of starch (Yonemoto et al., 2007). Zeng et al. (2014) showed that B-type starch granules with a size of ≤10 μm were more sensitive to temperature changes during the gelation process than A-type starch granules with a size of >10 μm. Thus, the effects of onset temperature, peak temperature and end temperature were greater on B-type starch granules than on A-type starch granules. Peterson and Fulcher (2001) showed that an increase in starch gelatinization characteristics was related to changes in starch composition and starch particle size distribution. Therefore, the higher the proportion of A-type starch granules, the lower the gelatinization characteristics of wheat starch. In the present study, correlation analysis revealed that the grain yield was significantly positively correlated with the contents of starch and its components, the proportion of B-type starch granules, starch gelatinization characteristics and starch thermodynamic characteristics; however, it was significantly negatively correlated with the proportion of A-type starch granules. The content of starch and its components was significantly positively correlated with starch gelatinization characteristics and starch thermodynamic characteristics. the proportion of A-type starch granules were significantly negatively correlated with grain yield, the proportion of B-type starch granules, content of starch and its components, starch gelatinization characteristics and starch thermodynamic characteristics. The content of wheat starch and its components was significantly positively correlated with starch gelatinization characteristics and starch thermodynamic characteristics.

## 5 Conclusions

In summary, weak light treatment post anthesis led to a significant reduction in the contents of starch and its components, including amylose and amylopectin, in soft wheat grains, thereby affecting grain number per spike and 1000-grain weight and ultimately resulting in decreased yield. Under the same treatment conditions, the effect of B-type starch granules on wheat starch granules was greater than that of A-type starch granules. In other words, the volume, surface area and number of B-type starch granules decreased, whereas those of A-type starch granules increased. The pasting properties of wheat, such as peak viscosity, were significantly reduced. Although the enthalpy parameters of wheat starch increased, the onset, peak and end temperatures significantly decreased, which affected the starch quality of wheat grains. Therefore, under conditions of weak light stress, an appropriate amount of N fertilizer should be applied to improve the 1000-grain weight of wheat, promote the absorption and utilization of N in wheat grains, improve the utilization efficiency of N fertilizer and form a good cultivation environment to alleviate the negative impact of weak light stress on wheat and increase the overall yield.

## Data Availability

The original contributions presented in the study are included in the article/supplementary material. Further inquiries can be directed to the corresponding authors.
